# The increasing expression of *GPX7* related to the malignant clinical features leading to poor prognosis of glioma patients

**DOI:** 10.1186/s41016-021-00235-3

**Published:** 2021-03-10

**Authors:** Jiawei Yao, Xin Chen, Zhendong Liu, Ruotian Zhang, Cheng Zhang, Quan Yang, Penglei Yao, Qiuyi Jiang, Jianing Wu, Shiguang Zhao

**Affiliations:** 1 Department of Neurosurgery, First Affiliated Hospital of Harbin Medical University, No. 23 Youzheng Street, Nangang District, Harbin, 150001 Heilongjiang Province China; 2 Key Colleges and Universities Laboratory of Neurosurgery in Heilongjiang Province, Harbin, 150001 Heilongjiang Province China; 3 Department of Neurosurgery, The Pinghu Hospital of Shenzhen University, Shenzhen, 518100 Guangdong Province China; 4 Institute of Neuroscience, Sino-Russian Medical Research Center, Harbin Medical University, Harbin, 150001 Heilongjiang Province China; 5 North Broward Preparatory School, 7600 Lyons Rd, Coconut Creek, FL 33073 USA

**Keywords:** *GPX7*, Glioma, Biomarker, Survival

## Abstract

**Background:**

Glioma is the most common malignant brain tumor in adults. The standard treatment scheme of glioma is surgical resection combined alternative radio- and chemotherapy. However, the outcome of glioma patients was unsatisfied. Here, we aimed to explore the molecular and biological function characteristics of GPX7 in glioma.

**Methods:**

The multidimensional data of glioma samples were downloaded from Chinese Glioma Genome Atlas (CGGA). RT-qPCR method was used to identify the expression status of GPX7. Kaplan–Meier curves and Cox regression analysis were used to explore the prognostic value of GPX7. Gene Set Enrichment Analysis (GSEA) was applied to investigate the GPX7-related functions in glioma.

**Results:**

The results indicated that the expression of *GPX7* in glioma was higher compared to that in normal brain tissue. Univariate and multivariate Cox regression analyses confirmed that the expression value of GPX7 was an independent prognostic factor in glioma. The GSEA analysis showed that *GPX7* was significantly enriched in the cell cycle pathway, ECM pathway, focal adhesion pathway, and toll-like receptor pathway.

**Conclusions:**

The *GPX7* was recommended as an independent risk factor for patients diagnosed with glioma for the first time and GPX7 could be potentially used as the therapy target in future. Furthermore, we attempted to explore a potential biomarker for improving the diagnosis and prognosis of patients with glioma.

**Supplementary Information:**

The online version contains supplementary material available at 10.1186/s41016-021-00235-3.

## Background

Glioma is a malignant tumor with the highest morbidity and mortality in the central nervous system, which represented up to 81% of central nervous system malignancies [1, 2]. The current standard treatment is surgical resection, combined radiotherapy and chemotherapy [3]. Although active treatments are taken, the overall survival (OS) and progression-free survival of glioma patients is still unsatisfying and unpredictable [4].. Therefore, it is imperative to identify the novel prognostic biomarkers for glioma.

Considerable studies have demonstrated that mRNA is involved in the molecular regulation mechanism of glioma. Thus, mRNA can be used as a potential biomarker to predict the overall survival time of glioma patients [5]. For instance, *LGALS3*, *L1CAM*, and *SCAMP3* were associated with the shorten OS of glioma patients by promoting the proliferation or other malignant tumor characteristics of glioma [6–8]. Despite previous studies partially revealed that the abnormal expression of mRNA is implicated in the occurrence and development of glioma via multiple mechanisms, the pathogenesis of these mRNAs remains limited due to the complex biological characteristics in gliomas. Therefore, the aim of this study was to explore a crucial mRNA for glioma to predict the prognosis of glioma patients.

Mammalian *GPX7* is a non-selenocysteine containing phospholipid peroxide glutathione peroxidase [9] and plays a significant role in maintaining redox status [10]. Therefore, the body avoids entering an oxidative stress state. Numerous studies have revealed that oxidative stress state exhibited an inhibitory effect on the pathological process of tumors. For example, miRNA-338-5p promotes oxidative stress by regulating the Hedgehog pathway and ultimately inhibited the proliferation of gliomas [11]. Moreover, inducing oxidative stress of C6 glioma cells was related with the promotion of cell cycle arrest and apoptosis [12]. However, whether *GPX7* was involved in the pathological process of tumors remains unknown. Peng et al. showed that *GPX7* was absent in esophageal adenocarcinoma and was associated with methylation of specific sites in the exon region of *GPX7*. Besides, the biological effect of overexpressing *GPX7* on inhibiting cell proliferation and promoting cell senescence has been verified in vitro experiments and in vivo models [13]. However, the expression of GPX7 was also reported as an oncogene. Nonetheless, recent studies have showed that overexpression of *GPX7* could predict malignant entities in liver cancer [14]. Hence, the role of *GPX7* in tumors remains controversial. So far, there is no relevant report of the function of *GPX7* in glioma. Thus, the role of *GPX7* in glioma, whether it could promote the pathological process of glioma or serves as a clinical therapeutic target, remained elusive.

Here, we investigated the molecular and clinical features of *GPX7* in glioma through bioinformatics analysis. Meanwhile, we attempted to reveal the GPX7-related pathological progress in gliomas. At the same time, we endeavored to establish a novel therapeutic target to improve the diagnosis and the prognosis of glioma.

## Methods

### RNA sequencing and clinic information from CGGA data repository

We downloaded the clinical and multidimensional data of more than 1000 Chinese glioma patients from the Chinese Glioma Genome Atlas (CGGA) (http://www.cgga.org.cn/). The data included the whole-exome sequencing (286), DNA methylation (159), mRNA sequencing (1018), mRNA microarray (301), microRNA microarray (198), and matched clinical data. In CGGA database, the RNA sequencing data and clinical information of 325 and 693 patients were obtained in two sessions. The sva package and limma package in R language were used to intersect the genes of the two data sets and merge the obtained data into one data set for subsequent analysis. Further, the glioma samples with incomplete clinical information were eliminated. And finally, 749 patients were enrolled in our study.

Gene expression profiling interactive analysis (GEPIA) database (http://gepia.cancer-pku.cn/) is a web server for gene expression profiling and interaction analysis of cancer and normal tissues. A total of 163 samples of glioblastoma, 518 samples of low-grade glioma, and 207 normal samples were enrolled in our study.

### GSEA

Gene Set Enrichment Analysis (GSEA) is a useful tool which could be used to analyze the functions and signal pathways of genes. The samples downloaded from the CGGA database were separated into high *GPX7*-expression group and low *GPX7*-expression group. A *p* value of less than 0.05 and an FDR value of less than 0.25 were significant.

### RT-qPCR

Total RNA was extracted from gliomas and normal control tissues by tri-Regent (Sigma, USA) (samples source: the First Affiliated Hospital of Harbin Medical University), RT-qPCR was performed using fast start universal SYBR Green Master (Rox) (Roche, Germany), -Δ CT reckoned the mRNA expression. The sequence of GAPDH was 5′-CAAGGTCATCACTGATGAACTTTG-3′ (F) and 5′-GTCCACCCTGTTGCTGTAG-3′ (R), and the primer sequence of *GPX7* was 5′-TCACAGACCACTACCGA-3′ (F) and 5′-CGGGGACACTACTCATTC-3′ (R).

### Statistical analysis

The transcriptome data from glioma samples from the CGGA database were comprehensively analyzed. Kaplan–Meier method and Cox regression model were used to exploring the prognostic value of GPX7 in glioma patients. Gene set enrichment analysis (GSEA) was used to predict the *GPX7-*related biological functions and pathways in gliomas. Co-expression analysis was used to detect the genes most related to the expression of *GPX7*.

## Results

### Clinical characteristics

The clinical information of gliomas, including PRS type, histology, grade, gender, age, radio status, chemotherapy, IDH mutation status, and 1p19q codeletion status, were shown in Table S1.

### High expression of *GPX7* shortens the overall survival time in glioma patients

**Fig. 1:**
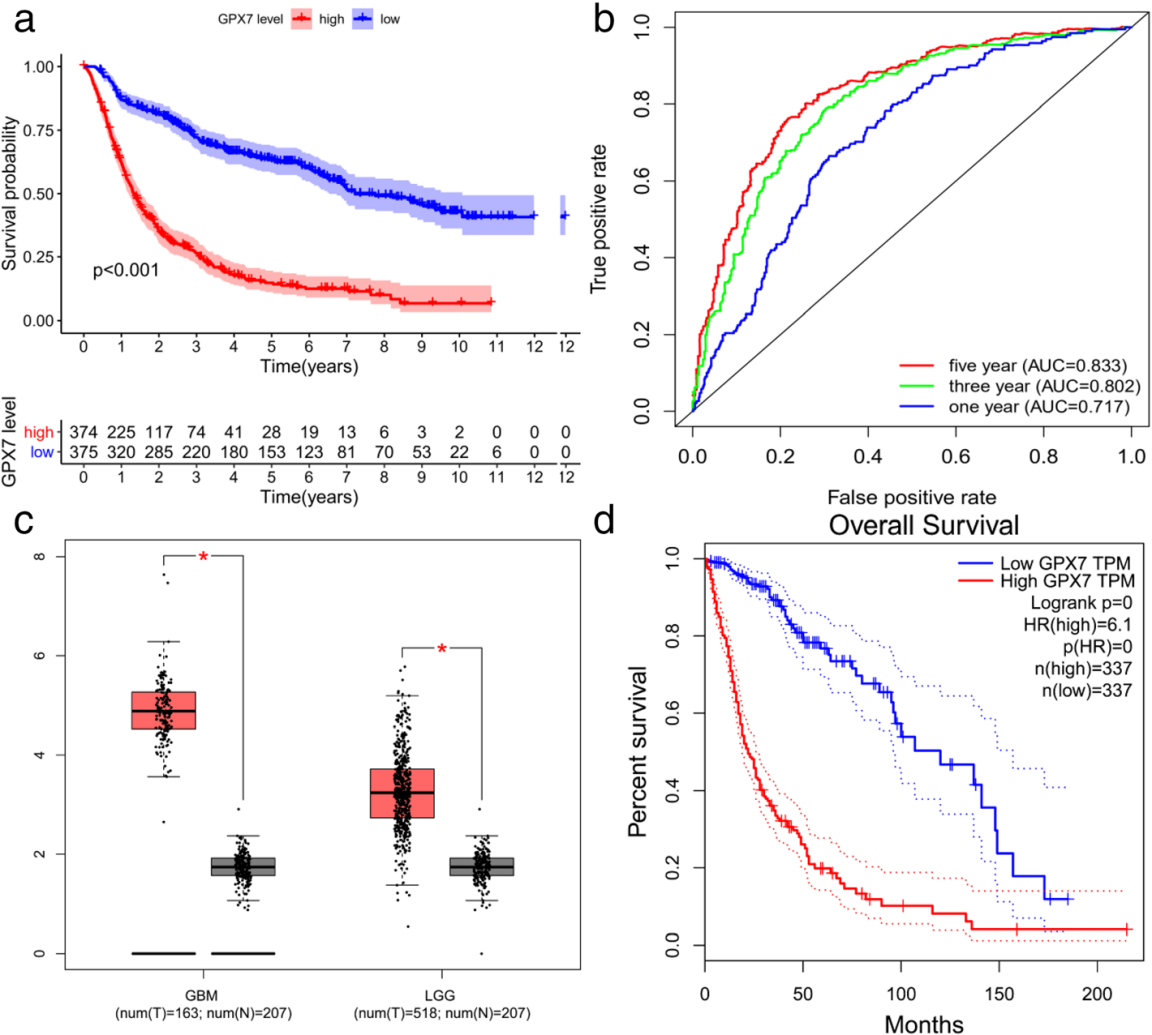
The Kaplan–Meier survival curve and The ROC curve. **a** Highly expressed *GPX7* leads to shorter overall survival time for patients with glioma. **b** ROC curve shows that using *GPX7* as a prognostic marker is valuable. **c** The expression of *GPX7* in GBM and LGG was higher than that in normal tissues in the GEPIA database. **d** Glioma patients with high *GPX7* expression have shorter survival times than those with low expression in the GEPIA database

To explore how *GPX7* affects glioma patients, we separated the obtained samples into high *GPX7* expression group and a low *GPX7* expression group. The survival package and survminer package were used to analyze the survival differences between two group. The five-year survival rate of the *GPX7* overexpression group was 14.7% (95% CI [0.1107–0.196]) while the 5-year survival rate of the *GPX7* low expression group was 63.3% (95% CI [0.583–0.689]) (Fig. 1a).

The receiver operating characteristic curve (ROC) was used to further verify whether the expression level of *GPX7* was reliable in predicting the survival time of patients. The AUC was more than 0.7 in the 1-year, 3-year, and 5-year ROC curve for the prognostic model (Fig. 1b). The area under the ROC curve (AUC) was more significant than 0.7, and which suggested that the GPX7 expression was reliable in predicting survival time.

Additionally, according to the GEPIA database, the expression of GPX7 was significantly higher in glioblastoma (GBM) and low-grade gliomas (LGG) than normal tissues. What is more, high *GPX7* expression remains associated with poor prognosis of glioma patients (*p* < 0.05) (Fig. 1c, d).

### Overexpressed *GPX7* as an independent risk factor for predicting the prognosis of glioma patients

**Fig. 2:**
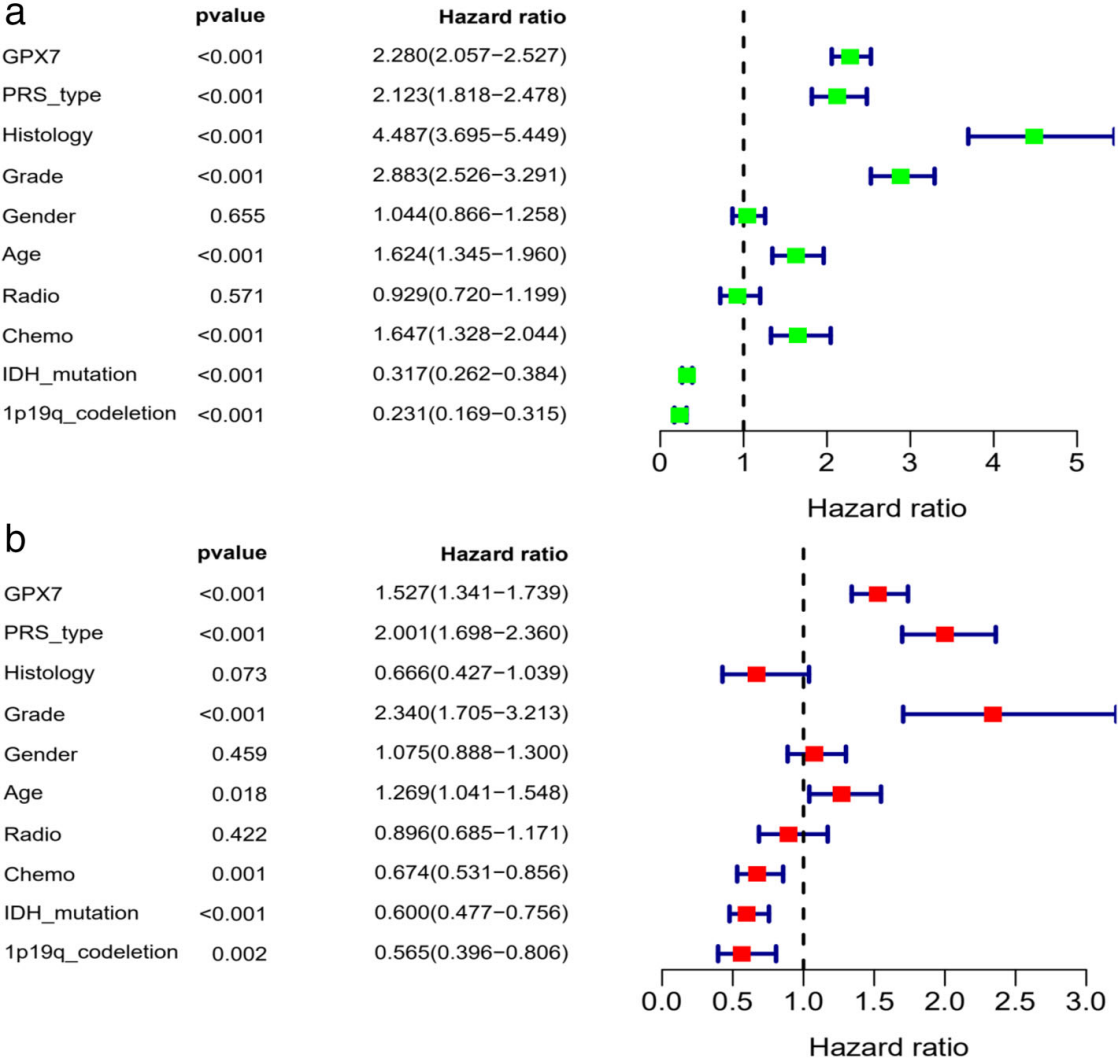
Relationship between clinical features and prognosis of patients with glioma. **a** Univariate analysis. **b** Multivariate analysis

Furthermore, to verify whether *GPX7* can be used as an independent risk factor in predicting the prognosis of glioma patients, the Cox regression model was used to assess *GPX7* expression and other clinical features. Univariate and multivariate Cox analysis confirmed that *GPX7* was an independent risk factor for glioma patients. The remaining clinical features associated with the OS of glioma patients are shown in Fig. 2.

### The relationship between *GPX7* and different clinical characteristics

**Fig. 3:**
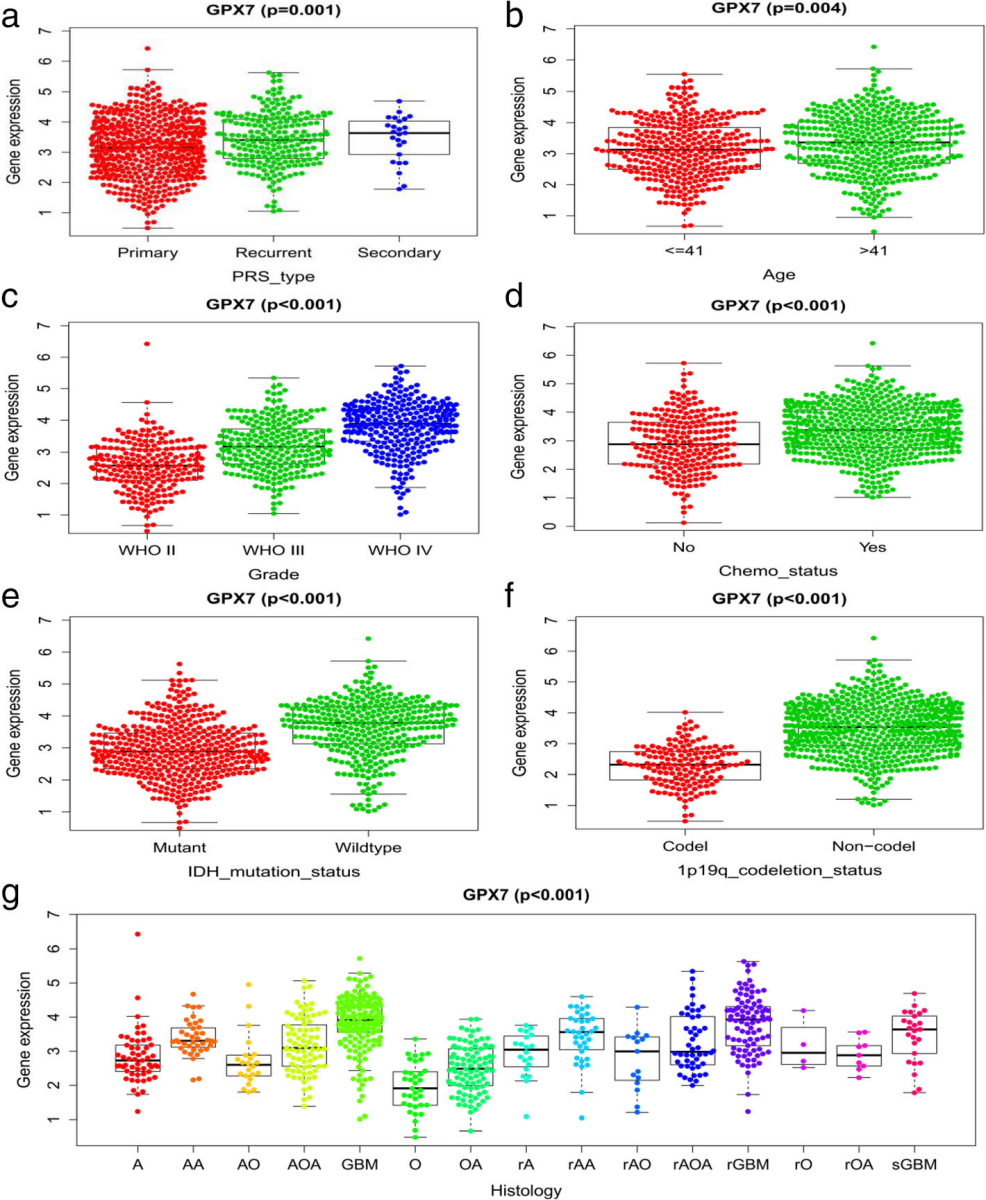
Relationship between *GPX7* and clinical characteristics. The expression of *GPX7* is positively correlated with **a** PRS type, **b** age, **c** grade, **d** chemotherapy, and **g** histology. The expression of *GPX7* is negatively correlated with **e** IDH mutation and **f** 1p/19q codeletion

Based on previous studies, the factors that might influence the prognosis of glioma patients were identified. Then, we used the Wilcox.test and Kruskal.test to investigate the relationship between GPX7 and clinical factors. The results revealed that the expression of *GPX7* was positively correlated with PRS type, history, grade, age, and chemotherapy, while it was negatively correlated with IDH mutation status and 1p19q codeletion status (Fig. 3a-g).

### Potential pathway of *GPX7* in regulating malignant biological behavior of glioma

**Fig. 4:**
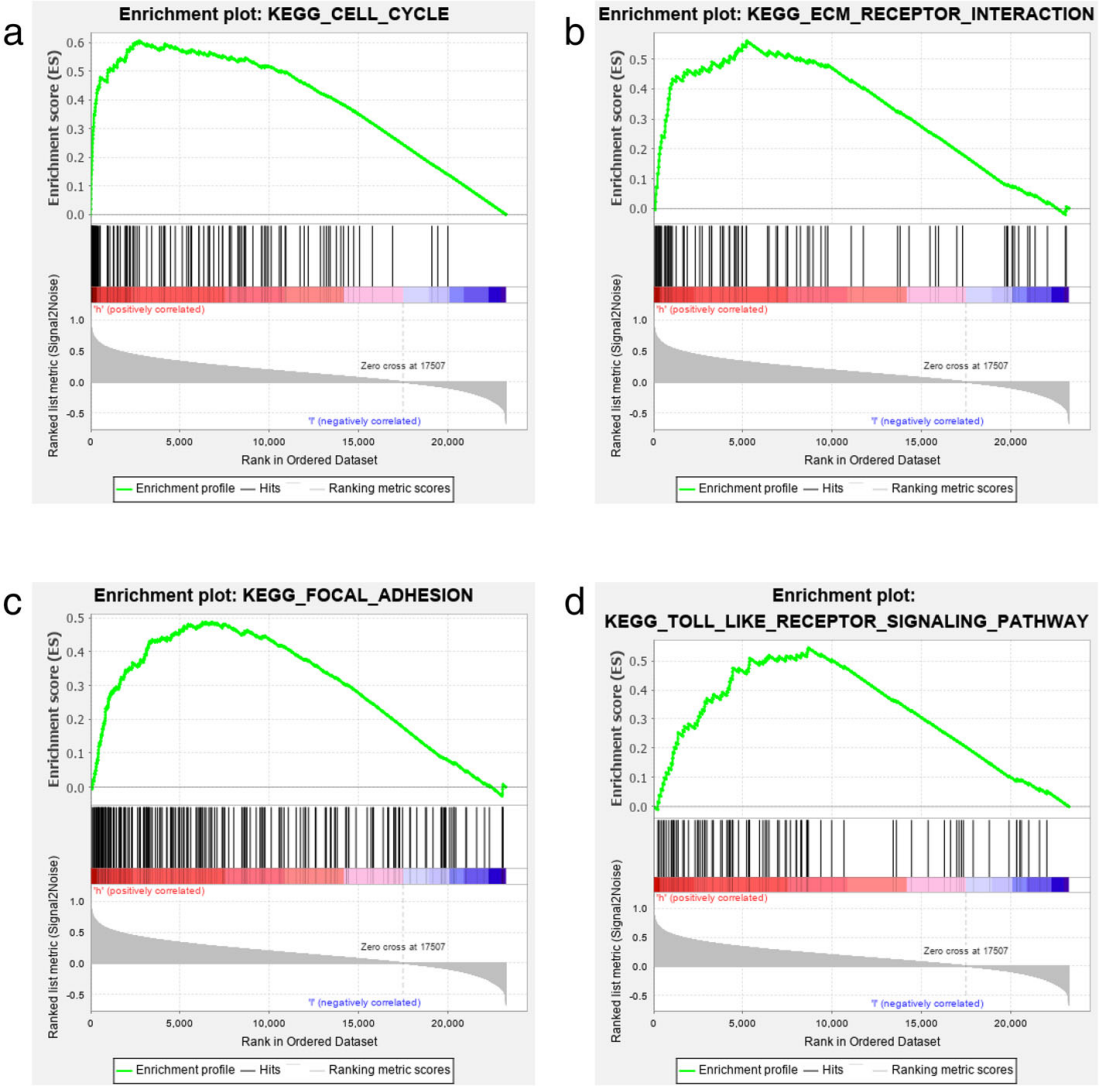
Significantly enriched pathways with GSEA. **a** Cell cycle pathway. **b** ECM pathway. **c** Focal adhesion pathway. **d** Toll-like receptor pathway

We used the GSEA method to identify the GPX7-related biological functions. Notably, GSEA is a ubiquitous bioinformatics analysis tool. This can help researchers identify the enrichment pathway of the target gene and indirectly understand the biological function of the target gene. Therefore, *GPX7* was divided into a high expression group and a low expression group. The results indicated that the GPX7 expression was related to multiple tumor-related pathways, including the cell cycle pathway, ECM pathway, focal adhesion pathway, and toll-like receptor pathway (Fig. 4a–d). These pathways were thus considered as potential strategies for *GPX7* to achieve its regulation in glioma.

### Co-expression analysis

**Fig. 5:**
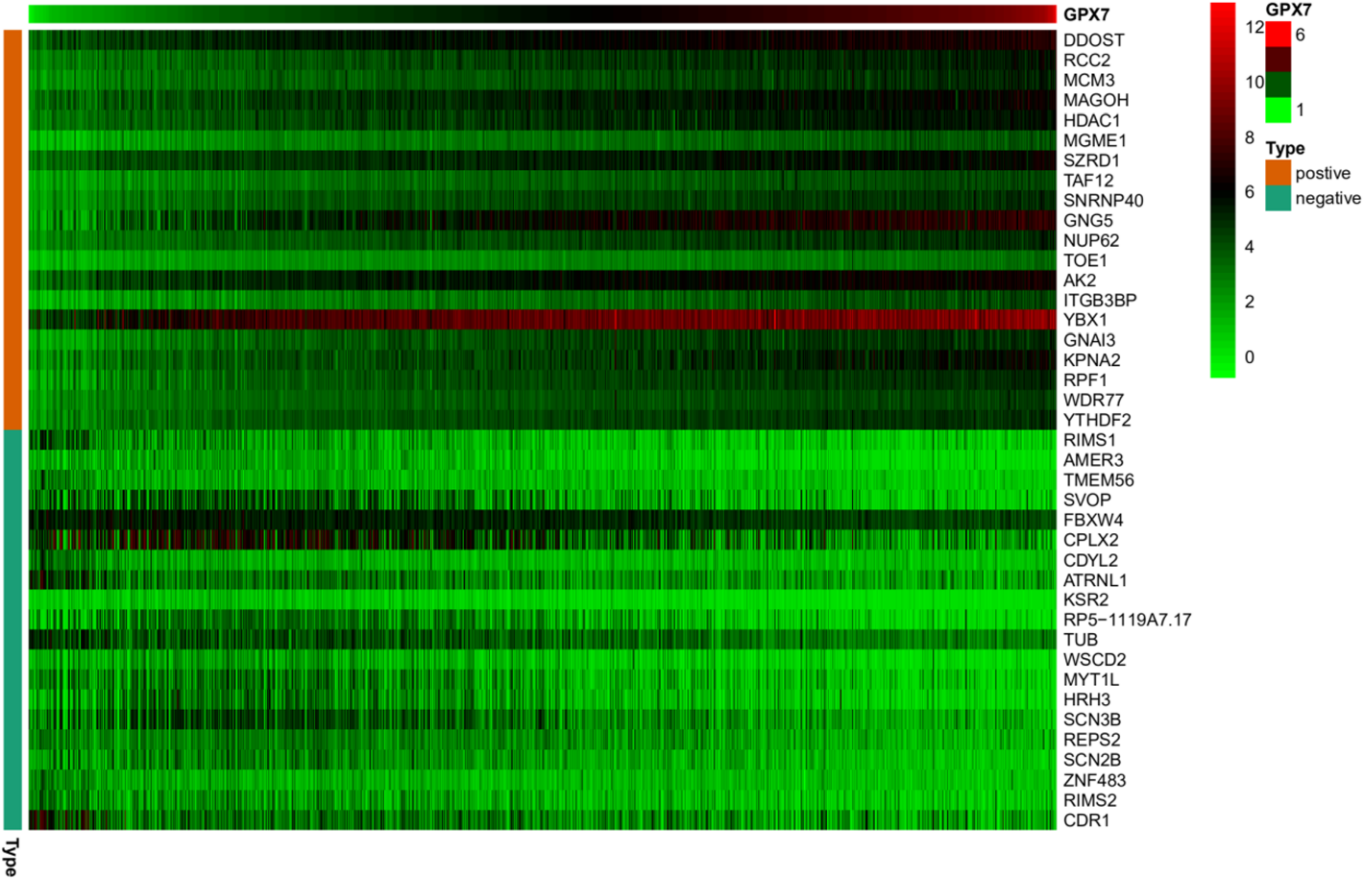
Gene expression heat map and correlations for *GPX7* co-expressed genes

**Fig. 6:**
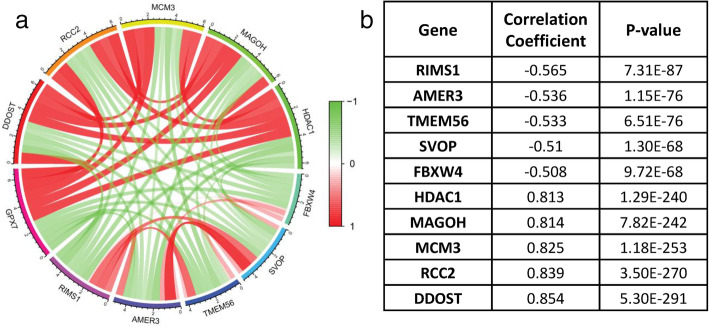
The co-expressed network of *GPX7*. **a** The circos plot was constructed by 10 genes most related to *GPX7* expression, including 5 positively related genes and 5 negatively related genes (*p* < 0.05). **b** Co-expression relationship of 10 genes in *GPX7*

To further clarify the role of *GPX7* in the pathophysiology of gliomas, we found the GPX7-related genes with the Pearson correlation test. Top five genes which were positively correlated with GPX7, including *DDOST*, *RCC2*, *MCM3*, *MAGOH*, and *HDAC1*, were selected. Meanwhile, the top five genes which were negatively correlated with GPX7, including *RIMS1*, *AMER3*, *TMEM56*, *SVOP*, and *FBXW4* were selected. As shown in Figs. 5 and 6, the result suggested that *GPX7* might promote the expression of genes that are positively correlated with its expression level or that these co-expressed positively related genes co-promote or inhibit specific pathophysiological processes with *GPX7* to promote the occurrence and development of glioma. Conversely, *GPX7* might inhibit the expression of genes that are negatively correlated, or the function between them is antagonistic.

### Validating *GPX7* expression level in glioma using RT-qPCR

**Fig. 7:**
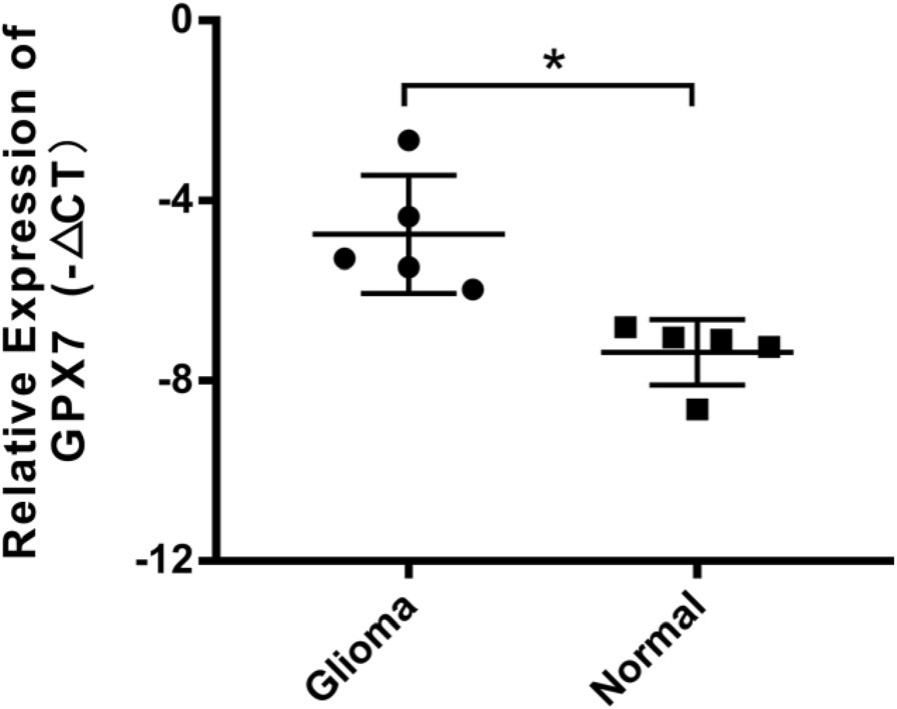
Detection of *GPX7* expression in gliomas by RT-qPCR

By analyzing the data from the CGGA database, it was concluded that *GPX7* is a novel oncogene, and its overexpression indicated a poor prognosis for gliomas. For further verification, 5 glioma samples and 5 normal brain tissue samples were used to verify the expression level of *GPX7* by RT-qPCR. As a result, the expression of *GPX7* in gliomas was higher compared to that in normal tissues (*p* < 0.05) (Fig. 7), which corroborated with the findings from previous analysis of the CGGA database.

## Discussion

Reports indicate that *GPX7* functions distinctly in different tumors. For instance, it is a tumor suppressor gene in esophageal adenocarcinoma but a carcinogenic gene in liver cancer [13, 14]. Nonetheless, the role of *GPX7* in glioma remains unknown. As such, to reveal the role of *GPX7* in glioma, this work first obtained RNA sequencing data of more than one thousand glioma patients from the CGGA database.

Through the correlation analysis between *GPX7* and OS of glioma patients, we discovered that the is the expression of GPX7 was negatively related with the OS in glioma patients. It is of pivotal importance to verify the reliability of *GPX7* in predicting the OS of glioma patients by the ROC curve. The overexpression of *GPX7* in gliomas was further validated by the GEPIA database and it was associated with poor prognosis. Nevertheless, the occurrence and development of glioma are induced by the imbalance between multiple carcinogenic and cancer suppressive factors; hence, several factors are implicated in this complex pathological process. Whether *GPX7* can be used as an independent risk factor for glioma patients remains unclear.

Univariate and multivariate analyses were performed by the Cox regression model. As a result, we found that *GPX7* could still be used as an independent risk factor in predicting the prognosis of glioma patients after excluding the influence of other factors. At the same time, our analysis demonstrated that other clinical characters, including PRS type, grade, age, chemotherapy, IDH mutation status, and 1p19q codeletion status, can also act as independent risk factors. Moreover, we uncovered that the expression level of *GPX7* is related to different clinical characteristics, thereby indicating that overexpression of *GPX7* might influence the malignant progress of these clinical characteristics via some potential mechanisms. Based on the above studies, we proved for the first time that *GPX7* could be used as a novel oncogene in predicting the prognosis of glioma patients. Nonetheless, the mechanism of *GPX7* in regulating the malignant behavior of glioma remains controversial.

GSEA analysis suggested that *GPX7* might involve in joining the pathophysiological process of glioma. The outcomes revealed some significantly enriched pathways, including cell cycle pathway, ECM pathway, focal adhesion pathway, and toll-like receptor pathway, Catchin flavonoids inhibited glioma by blocking cell cycle signaling pathways [15]. The invasiveness of glioma cells can be achieved by selectively interacting with the extracellular matrix (ECM) components [16]. Mda-9/syntenin promotes glioma migration through focal adhesion kinase (FAK) in the focal adhesion signaling pathway [17]. The toll-like receptor family is closely related to cancer. Toll-like receptor 4 promotes glioma proliferation by activating the NF-kappa B signaling pathway [18]. Toll-like receptor 2 regulates glioma invasion by promoting the expression of matrix metalloproteinases [19]. The above pathways promote the pathological process of glioma through proliferation, migration, invasion, and other malignant biological behaviors of glioma cells. The enrichment of *GPX7* in these pathways revealed that glioma might participate in the regulation via various complex pathways. Despite reaching a preliminary conclusion on how *GPX7* regulates glioma by GSEA analysis, the biological behavior of glioma cannot be fully regulated by a single factor due to the complex pathological process of glioma.

Therefore, we screened 10 genes having the highest correlation with *GPX7* expression by co-expression analysis, among which 5 genes had a positive correlation, including *DDOST*, *RCC2*, *MCM3*, *MAGOH*, and *HDAC1*. The pathophysiological processes regulated by these genes were closely related to tumors. The high expression of *DDOST* in colon adenocarcinoma promotes the proliferation of tumor cells [20]. The expression of *RCC2* in ER-positive breast induces the expression of IGF-1 and leads to the malignant behavior of tumor cells, including proliferation, invasion, etc. [21]. *MCM3* is an independent risk factor for invasive ductal carcinoma [22]. *MAGOH* regulates melanoma production by controlling the proliferation of melanocyte [23]. *HDAC1* promotes liver cancer metastasis via the FAM99A-miR92a signaling pathway [24]. Additionally, the 5 negatively related genes include *RIMS1*, *AMER3*, *TMEM56*, *SVOP*, and *FBXW4*. Therefore, the occurrence and development of glioma is a significantly complex biological process, and *GPX7* interacts with various oncogenes and tumor suppressor genes to participate in the network of glioma regulation. *GPX7* is considered as a novel molecular marker that predicts the prognosis of glioma patients, with high validity and reliability.

For the first time, through analysis of multidimensional data, we propose that *GPX7* can be used as a novel oncogene to shorten the prognosis of glioma patients. Nonetheless, this study has some shortcomings. Firstly, the CGGA database only enrolled more than 1000 high-throughput data of glioma patients without non-glioma individuals. Therefore, all the findings were based on the comparison between the high- and low-expression of the *GPX7* group. However, we found that the high expression of *GPX7* accounts for malignant progression of glioma by comparing the overall survival time between the two groups and verifying its credibility via ROC curves. The expression of *GPX7* was validated in glioma and normal brain tissues by RT-qPCR to compensate for the insufficiency of sample information in the database. Consequently, we found that *GPX7* was highly expressed in glioma. Notably, this was in line with our previous findings.

## Conclusion

For the first time, the *GPX7* was reported as a carcinogenic gene in glioma, and our results revealed the correlation between gliomas and malignant clinical features. Moreover, our findings provided a new diagnostic and prognostic marker for study in future.

## Supplementary Information

**Additional file 1: Table S1.** Characteristics of patients with glioma based on CGGA. Description of data: Clinical information of the samples we used for analysis.

## Data Availability

All data generated or analyzed during this study are included in this published article.

## Abbreviations

CGGA: The Chinese Glioma Genome Atlas
GEPIA: Gene Expression Profiling Interactive Analysis
OS: Overall survival
GSEA: Gene Set Enrichment Analysis
ROC: Receiver operating characteristic curve
AUC: Area under the ROC curve
